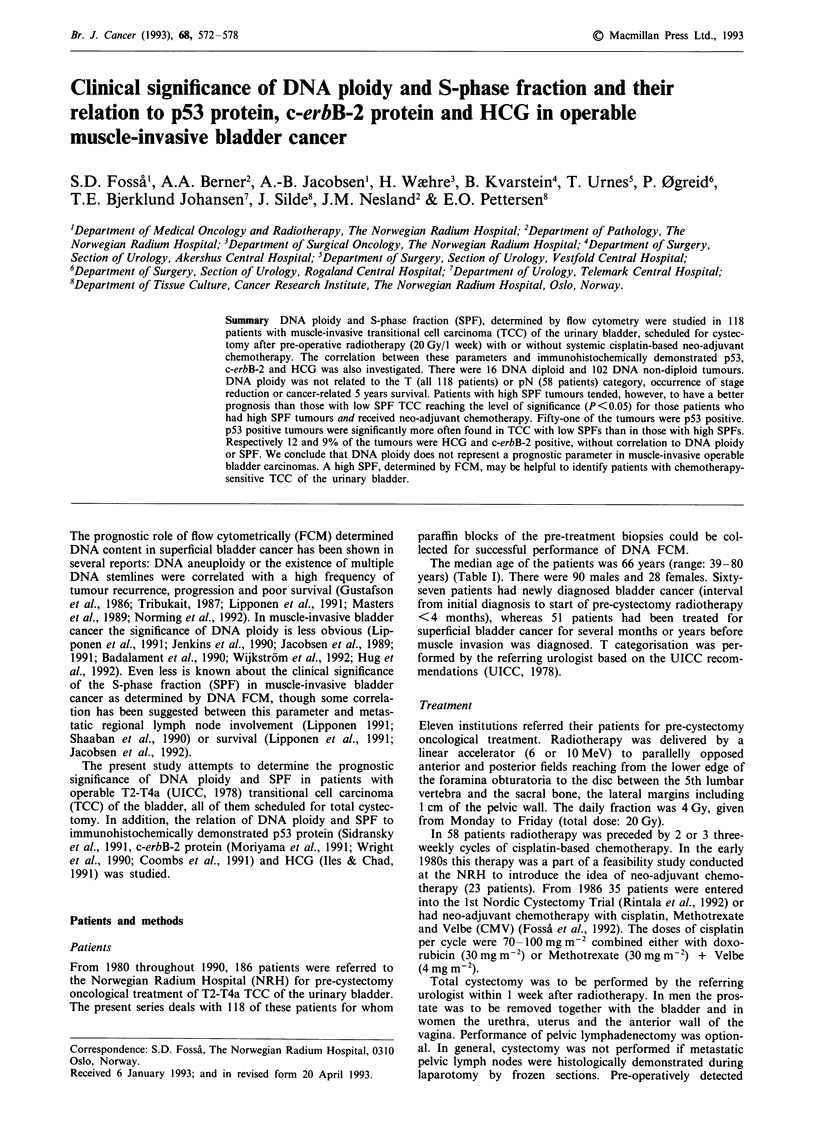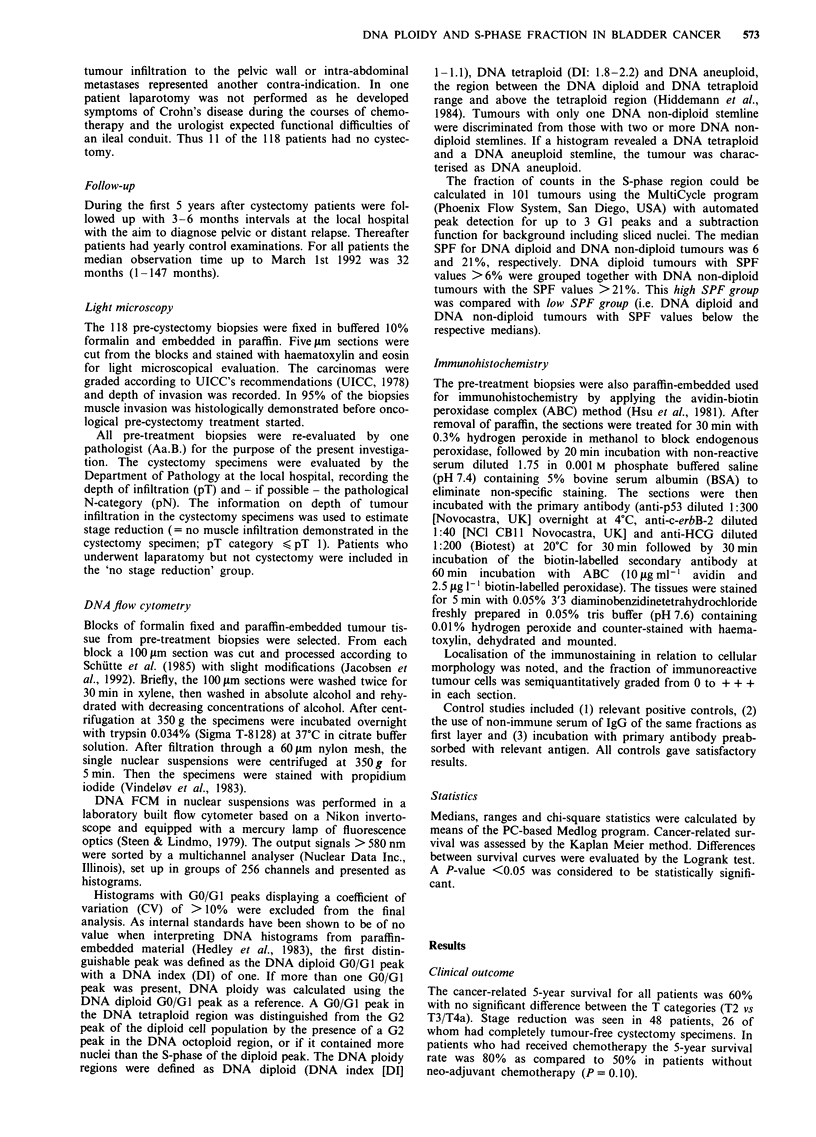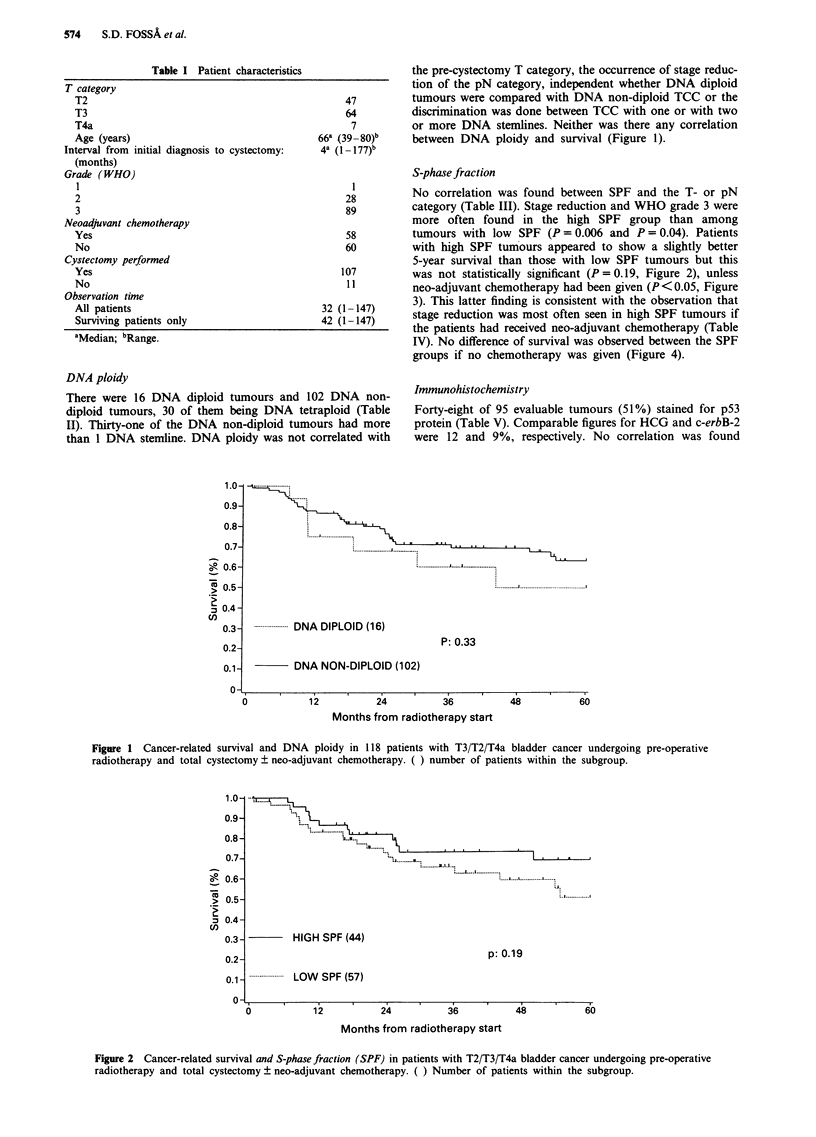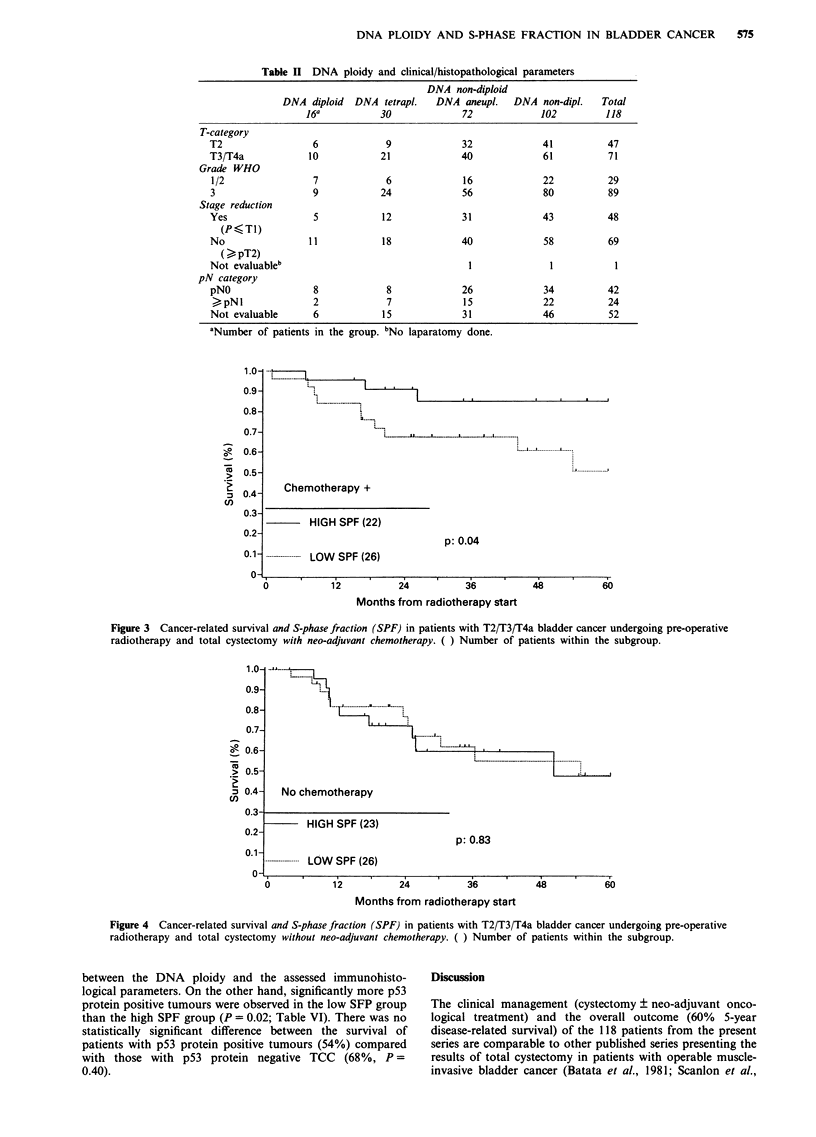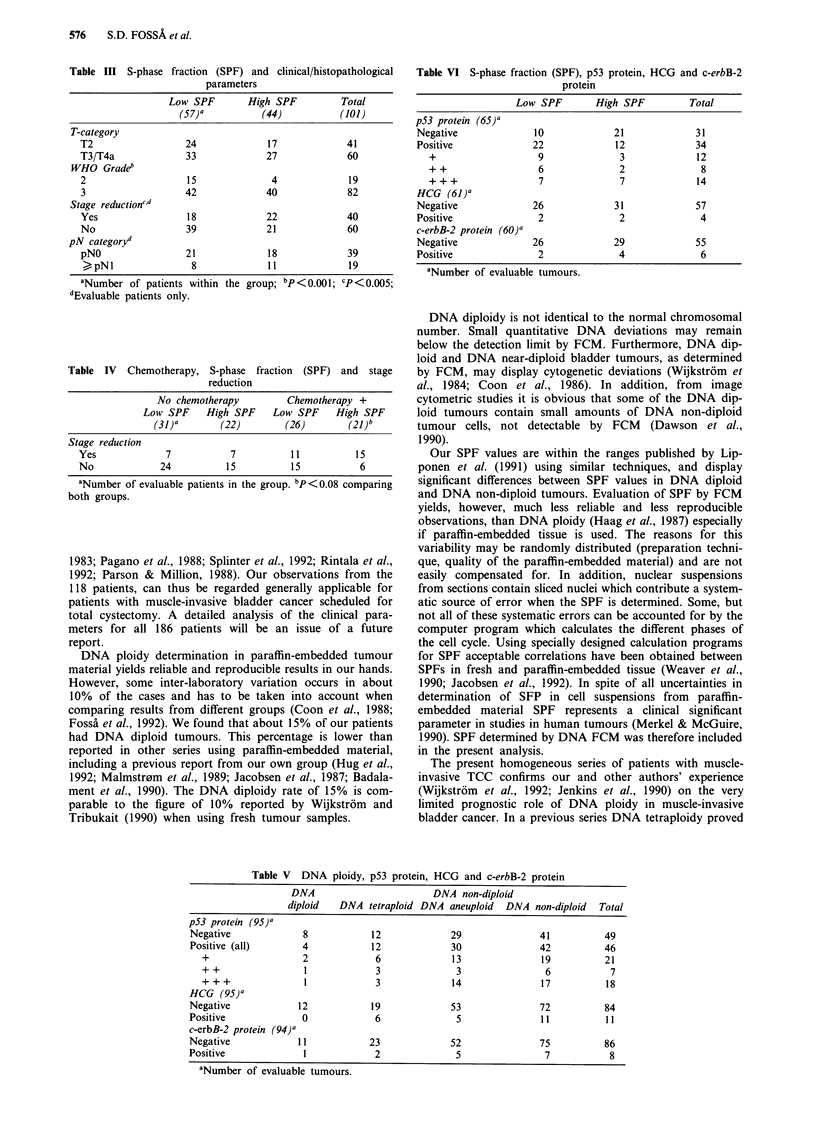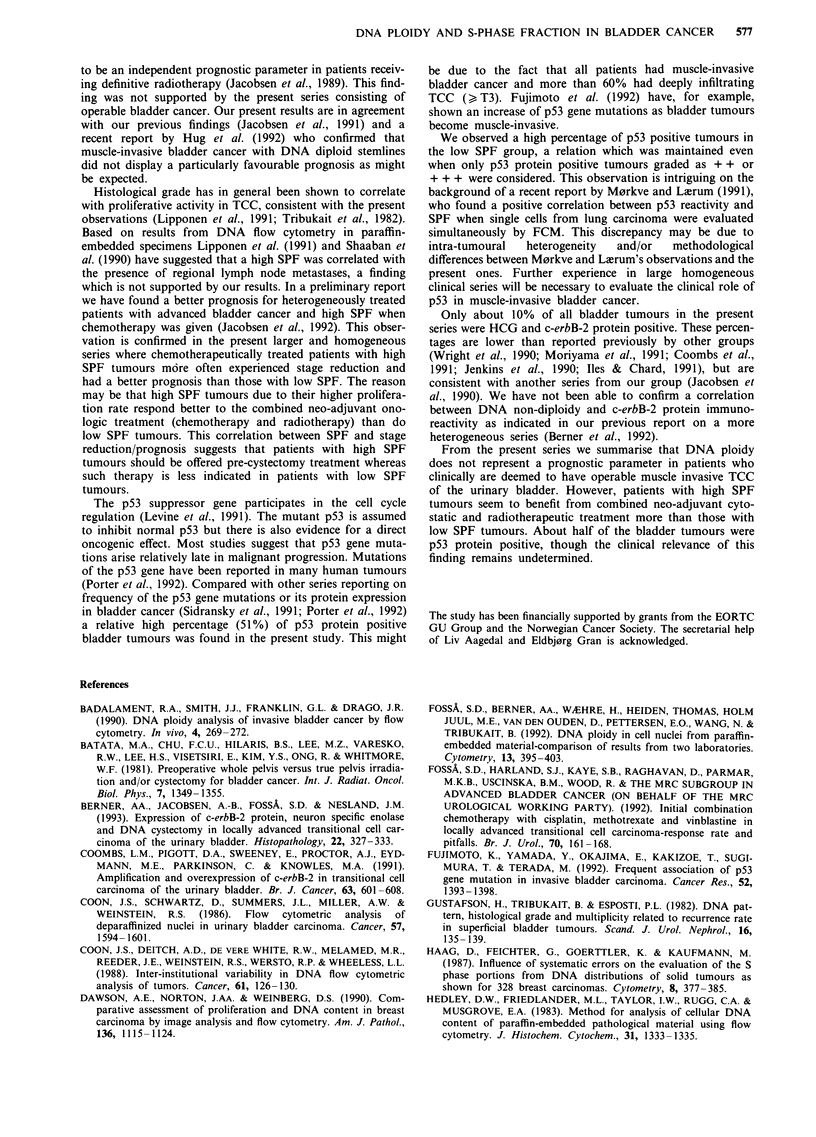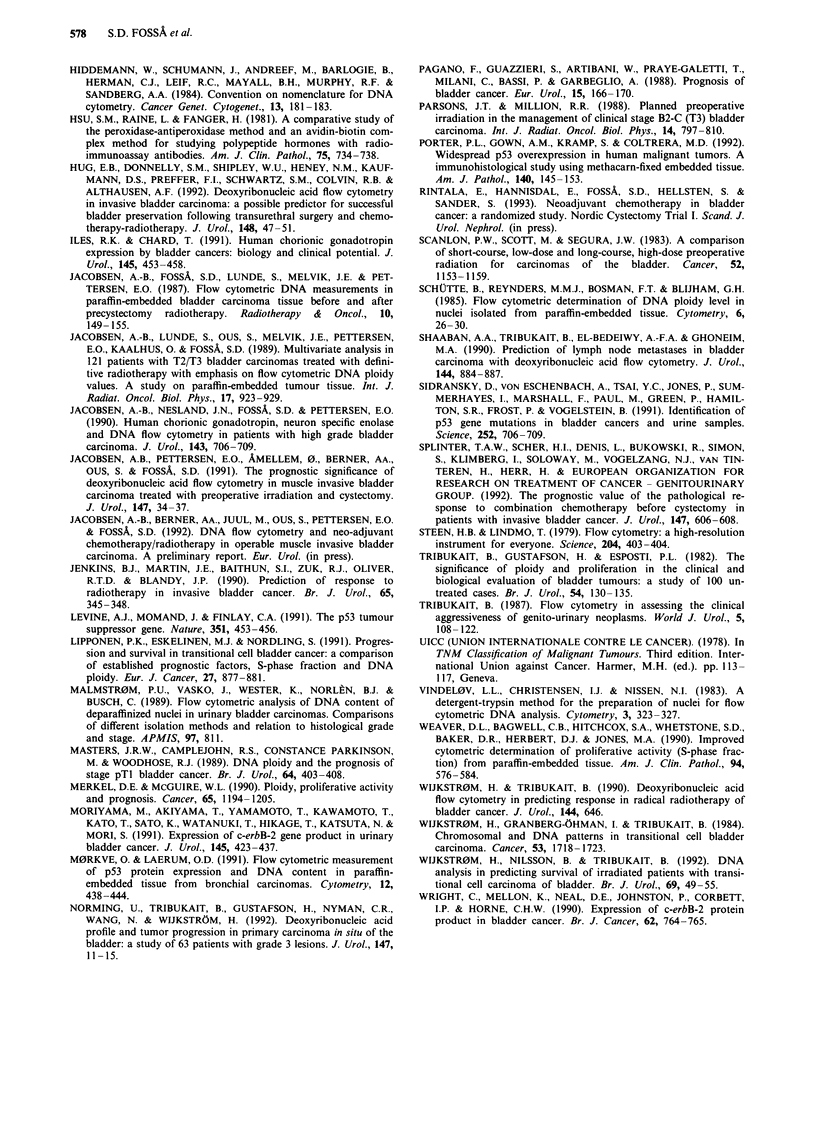# Clinical significance of DNA ploidy and S-phase fraction and their relation to p53 protein, c-erbB-2 protein and HCG in operable muscle-invasive bladder cancer.

**DOI:** 10.1038/bjc.1993.388

**Published:** 1993-09

**Authors:** S. D. Fosså, A. A. Berner, A. B. Jacobsen, H. Waehre, B. Kvarstein, T. Urnes, P. Ogreid, T. E. Johansen, J. Silde, J. M. Nesland

**Affiliations:** Department of Medical Oncology and Radiotherapy, Norwegian Radium Hospital, Oslo.

## Abstract

DNA ploidy and S-phase fraction (SPF), determined by flow cytometry were studied in 118 patients with muscle-invasive transitional cell carcinoma (TCC) of the urinary bladder, scheduled for cystectomy after pre-operative radiotherapy (20 Gy/1 week) with or without systemic cisplatin-based neo-adjuvant chemotherapy. The correlation between these parameters and immunohistochemically demonstrated p53, c-erbB-2 and HCG was also investigated. There were 16 DNA diploid and 102 DNA non-diploid tumours. DNA ploidy was not related to the T (all 118 patients) or pN (58 patients) category, occurrence of stage reduction or cancer-related 5 years survival. Patients with high SPF tumours tended, however, to have a better prognosis than those with low SPF TCC reaching the level of significance (P < 0.05) for those patients who had high SPF tumours and received neo-adjuvant chemotherapy. Fifty-one of the tumours were p53 positive. p53 positive tumours were significantly more often found in TCC with low SPFs than in those with high SPFs. Respectively 12 and 9% of the tumours were HCG and c-erbB-2 positive, without correlation to DNA ploidy or SPF. We conclude that DNA ploidy does not represent a prognostic parameter in muscle-invasive operable bladder carcinomas. A high SPF, determined by FCM, may be helpful to identify patients with chemotherapy-sensitive TCC of the urinary bladder.


 © Macmillan Press Ltd., 1993

### Clinical significance of DNA ploidy and S-phase fraction and their relation to p53 protein, c-*erb*B-2 protein and HCG in operable muscle-invasive bladder cancer

S.D. Fosså[1], A.A. Berner[2], A.-B. Jacobsen[1], H. Wæhre[3], B. Kvarstein[4], T. Urnes[5], P. Øgreid[6], T.E. Bjerklund Johansen[7], J. Silde[8], J.M. Nesland[2] & E.O. Pettersen[8]

[1]*Department of Medical Oncology and Radiotherapy, The Norwegian Radium Hospital;* [2]*Department of Pathology, The Norwegian Radium Hospital;* [3]*Department of Surgical Oncology, The Norwegian Radium Hospital;* [4]*Department of Surgery, Section of Urology, Akershus Central Hospital;* [5]*Department of Surgery, Section of Urology, Vestfold Central Hospital;* [6]*Department of Surgery, Section of Urology, Rogaland Central Hospital;* [7]*Department of Urology, Telemark Central Hospital;* [8]*Department of Tissue Culture, Cancer Research Institute, The Norwegian Radium Hospital, Oslo, Norway.*

**Summary** DNA ploidy and S-phase fraction (SPF), determined by flow cytometry were studied in 118 patients with muscle-invasive transitional cell carcinoma (TCC) of the urinary bladder, scheduled for cystectomy after pre-operative radiotherapy (20 Gy/1 week) with or without systemic cisplatin-based neo-adjuvant chemotherapy. The correlation between these parameters and immunohistochemically demonstrated p53, c-*erb*B-2 and HCG was also investigated. There were 16 DNA diploid and 102 DNA non-diploid tumours. DNA ploidy was not related to the T (all 118 patients) or pN (58 patients) category, occurrence of stage reduction or cancer-related 5 years survival. Patients with high SPF tumours tended, however, to have a better prognosis than those with low SPF TCC reaching the level of significance ($P < 0.05$) for those patients who had high SPF tumours *and* received neo-adjuvant chemotherapy. Fifty-one of the tumours were p53 positive. p53 positive tumours were significantly more often found in TCC with low SPFs than in those with high SPFs. Respectively 12 and 9% of the tumours were HCG and c-*erb*B-2 positive, without correlation to DNA ploidy or SPF. We conclude that DNA ploidy does not represent a prognostic parameter in muscle-invasive operable bladder carcinomas. A high SPF, determined by FCM, may be helpful to identify patients with chemotherapy-sensitive TCC of the urinary bladder.

The prognostic role of flow cytometrically (FCM) determined DNA content in superficial bladder cancer has been shown in several reports: DNA aneuploidy or the existence of multiple DNA stemlines were correlated with a high frequency of tumour recurrence, progression and poor survival (Gustafson *et al.*, 1986; Tribukait, 1987; Lipponen *et al.*, 1991; Masters *et al.*, 1989; Norming *et al.*, 1992). In muscle-invasive bladder cancer the significance of DNA ploidy is less obvious (Lipponen *et al.*, 1991; Jenkins *et al.*, 1990; Jacobsen *et al.*, 1989; 1991; Badalament *et al.*, 1990; Wijkström *et al.*, 1992; Hug *et al.*, 1992). Even less is known about the clinical significance of the S-phase fraction (SPF) in muscle-invasive bladder cancer as determined by DNA FCM, though some correlation has been suggested between this parameter and metastatic regional lymph node involvement (Lipponen 1991; Shaaban *et al.*, 1990) or survival (Lipponen *et al.*, 1991; Jacobsen *et al.*, 1992).

The present study attempts to determine the prognostic significance of DNA ploidy and SPF in patients with operable T2-T4a (UICC, 1978) transitional cell carcinoma (TCC) of the bladder, all of them scheduled for total cystectomy. In addition, the relation of DNA ploidy and SPF to immunohistochemically demonstrated p53 protein (Sidransky *et al.*, 1991, c-*erb*B-2 protein (Moriyama *et al.*, 1991; Wright *et al.*, 1990; Coombs *et al.*, 1991) and HCG (Iles & Chad, 1991) was studied.

### Patients and methods

### *Patients*

From 1980 throughout 1990, 186 patients were referred to the Norwegian Radium Hospital (NRH) for pre-cystectomy oncological treatment of T2-T4a TCC of the urinary bladder. The present series deals with 118 of these patients for whom paraffin blocks of the pre-treatment biopsies could be collected for successful performance of DNA FCM.

The median age of the patients was 66 years (range: 39-80 years) (Table I). There were 90 males and 28 females. Sixty-seven patients had newly diagnosed bladder cancer (interval from initial diagnosis to start of pre-cystectomy radiotherapy <4 months), whereas 51 patients had been treated for superficial bladder cancer for several months or years before muscle invasion was diagnosed. T categorisation was performed by the referring urologist based on the UICC recommendations (UICC, 1978).

### *Treatment*

Eleven institutions referred their patients for pre-cystectomy oncological treatment. Radiotherapy was delivered by a linear accelerator (6 or 10 MeV) to parallelly opposed anterior and posterior fields reaching from the lower edge of the foramina obturatoria to the disc between the 5th lumbar vertebra and the sacral bone, the lateral margins including 1 cm of the pelvic wall. The daily fraction was 4 Gy, given from Monday to Friday (total dose: 20 Gy).

In 58 patients radiotherapy was preceded by 2 or 3 three-weekly cycles of cisplatin-based chemotherapy. In the early 1980s this therapy was a part of a feasibility study conducted at the NRH to introduce the idea of neo-adjuvant chemotherapy (23 patients). From 1986 35 patients were entered into the 1st Nordic Cystectomy Trial (Rintala *et al.*, 1992) or had neo-adjuvant chemotherapy with cisplatin, Methotrexate and Velbe (CMV) (Fosså *et al.*, 1992). The doses of cisplatin per cycle were 70-100 mg m$^{-2}$ combined either with doxorubicin (30 mg m$^{-2}$) or Methotrexate (30 mg m$^{-2}$) + Velbe (4 mg m$^{-2}$).

Total cystectomy was to be performed by the referring urologist within 1 week after radiotherapy. In men the prostate was to be removed together with the bladder and in women the urethra, uterus and the anterior wall of the vagina. Performance of pelvic lymphadenectomy was optional. In general, cystectomy was not performed if metastatic pelvic lymph nodes were histologically demonstrated during laparotomy by frozen sections. Pre-operatively detected

Correspondence: S.D. Fosså, The Norwegian Radium Hospital, 0310 Oslo, Norway.
Received 6 January 1993; and in revised form 20 April 1993.

tumour infiltration to the pelvic wall or intra-abdominal metastases represented another contra-indication. In one patient laparotomy was not performed as he developed symptoms of Crohn's disease during the courses of chemotherapy and the urologist expected functional difficulties of an ileal conduit. Thus 11 of the 118 patients had no cystectomy.

*Follow-up*

During the first 5 years after cystectomy patients were followed up with 3–6 months intervals at the local hospital with the aim to diagnose pelvic or distant relapse. Thereafter patients had yearly control examinations. For all patients the median observation time up to March 1st 1992 was 32 months (1–147 months).

*Light microscopy*

The 118 pre-cystectomy biopsies were fixed in buffered 10% formalin and embedded in paraffin. Five μm sections were cut from the blocks and stained with haematoxylin and eosin for light microscopical evaluation. The carcinomas were graded according to UICC's recommendations (UICC, 1978) and depth of invasion was recorded. In 95% of the biopsies muscle invasion was histologically demonstrated before oncological pre-cystectomy treatment started.

All pre-treatment biopsies were re-evaluated by one pathologist (Aa.B.) for the purpose of the present investigation. The cystectomy specimens were evaluated by the Department of Pathology at the local hospital, recording the depth of infiltration (pT) and – if possible – the pathological N-category (pN). The information on depth of tumour infiltration in the cystectomy specimens was used to estimate stage reduction (= no muscle infiltration demonstrated in the cystectomy specimen; pT category $\leqslant$ pT 1). Patients who underwent laparatomy but not cystectomy were included in the 'no stage reduction' group.

*DNA flow cytometry*

Blocks of formalin fixed and paraffin-embedded tumour tissue from pre-treatment biopsies were selected. From each block a 100 μm section was cut and processed according to Schütte *et al.* (1985) with slight modifications (Jacobsen *et al.*, 1992). Briefly, the 100 μm sections were washed twice for 30 min in xylene, then washed in absolute alcohol and rehydrated with decreasing concentrations of alcohol. After centrifugation at 350 g the specimens were incubated overnight with trypsin 0.034% (Sigma T-8128) at 37°C in citrate buffer solution. After filtration through a 60 μm nylon mesh, the single nuclear suspensions were centrifuged at 350 *g* for 5 min. Then the specimens were stained with propidium iodide (Vindeløv *et al.*, 1983).

DNA FCM in nuclear suspensions was performed in a laboratory built flow cytometer based on a Nikon invertoscope and equipped with a mercury lamp of fluorescence optics (Steen & Lindmo, 1979). The output signals $>$580 nm were sorted by a multichannel analyser (Nuclear Data Inc., Illinois), set up in groups of 256 channels and presented as histograms.

Histograms with G0/G1 peaks displaying a coefficient of variation (CV) of $>$10% were excluded from the final analysis. As internal standards have been shown to be of no value when interpreting DNA histograms from paraffin-embedded material (Hedley *et al.*, 1983), the first distinguishable peak was defined as the DNA diploid G0/G1 peak with a DNA index (DI) of one. If more than one G0/G1 peak was present, DNA ploidy was calculated using the DNA diploid G0/G1 peak as a reference. A G0/G1 peak in the DNA tetraploid region was distinguished from the G2 peak of the diploid cell population by the presence of a G2 peak in the DNA octoploid region, or if it contained more nuclei than the S-phase of the diploid peak. The DNA ploidy regions were defined as DNA diploid (DNA index [DI] 1–1.1), DNA tetraploid (DI: 1.8–2.2) and DNA aneuploid, the region between the DNA diploid and DNA tetraploid range and above the tetraploid region (Hiddemann *et al.*, 1984). Tumours with only one DNA non-diploid stemline were discriminated from those with two or more DNA non-diploid stemlines. If a histogram revealed a DNA tetraploid and a DNA aneuploid stemline, the tumour was characterised as DNA aneuploid.

The fraction of counts in the S-phase region could be calculated in 101 tumours using the MultiCycle program (Phoenix Flow System, San Diego, USA) with automated peak detection for up to 3 G1 peaks and a subtraction function for background including sliced nuclei. The median SPF for DNA diploid and DNA non-diploid tumours was 6 and 21%, respectively. DNA diploid tumours with SPF values $>$6% were grouped together with DNA non-diploid tumours with the SPF values $>$21%. This ***high SPF group*** was compared with ***low SPF group*** (i.e. DNA diploid and DNA non-diploid tumours with SPF values below the respective medians).

*Immunohistochemistry*

The pre-treatment biopsies were also paraffin-embedded used for immunohistochemistry by applying the avidin-biotin peroxidase complex (ABC) method (Hsu *et al.*, 1981). After removal of paraffin, the sections were treated for 30 min with 0.3% hydrogen peroxide in methanol to block endogenous peroxidase, followed by 20 min incubation with non-reactive serum diluted 1.75 in 0.001 M phosphate buffered saline (pH 7.4) containing 5% bovine serum albumin (BSA) to eliminate non-specific staining. The sections were then incubated with the primary antibody (anti-p53 diluted 1:300 [Novocastra, UK] overnight at 4°C, anti-c-*erb*B-2 diluted 1:40 [NCl CB11 Novocastra, UK] and anti-HCG diluted 1:200 (Biotest) at 20°C for 30 min followed by 30 min incubation of the biotin-labelled secondary antibody at 60 min incubation with ABC (10 $\mu g\ ml^{-1}$ avidin and 2.5 $\mu g\ l^{-1}$ biotin-labelled peroxidase). The tissues were stained for 5 min with 0.05% 3'3 diaminobenzidinetetrahydrochloride freshly prepared in 0.05% tris buffer (pH 7.6) containing 0.01% hydrogen peroxide and counter-stained with haematoxylin, dehydrated and mounted.

Localisation of the immunostaining in relation to cellular morphology was noted, and the fraction of immunoreactive tumour cells was semiquantitatively graded from 0 to + + + in each section.

Control studies included (1) relevant positive controls, (2) the use of non-immune serum of IgG of the same fractions as first layer and (3) incubation with primary antibody preabsorbed with relevant antigen. All controls gave satisfactory results.

*Statistics*

Medians, ranges and chi-square statistics were calculated by means of the PC-based Medlog program. Cancer-related survival was assessed by the Kaplan Meier method. Differences between survival curves were evaluated by the Logrank test. A *P*-value $<$0.05 was considered to be statistically significant.

### Results

*Clinical outcome*

The cancer-related 5-year survival for all patients was 60% with no significant difference between the T categories (T2 *vs* T3/T4a). Stage reduction was seen in 48 patients, 26 of whom had completely tumour-free cystectomy specimens. In patients who had received chemotherapy the 5-year survival rate was 80% as compared to 50% in patients without neo-adjuvant chemotherapy ($P = 0.10$).

**Table I** Patient characteristics

| | |
|---|---|
| *T category* | |
| T2 | 47 |
| T3 | 64 |
| T4a | 7 |
| Age (years) | 66[a] (39–80)[b] |
| Interval from initial diagnosis to cystectomy: (months) | 4[a] (1–177)[b] |
| *Grade (WHO)* | |
| 1 | 1 |
| 2 | 28 |
| 3 | 89 |
| *Neoadjuvant chemotherapy* | |
| Yes | 58 |
| No | 60 |
| *Cystectomy performed* | |
| Yes | 107 |
| No | 11 |
| *Observation time* | |
| All patients | 32 (1–147) |
| Surviving patients only | 42 (1–147) |

[a]Median; [b]Range.

*DNA ploidy*

There were 16 DNA diploid tumours and 102 DNA non-diploid tumours, 30 of them being DNA tetraploid (Table II). Thirty-one of the DNA non-diploid tumours had more than 1 DNA stemline. DNA ploidy was not correlated with the pre-cystectomy T category, the occurrence of stage reduction of the pN category, independent whether DNA diploid tumours were compared with DNA non-diploid TCC or the discrimination was done between TCC with one or with two or more DNA stemlines. Neither was there any correlation between DNA ploidy and survival (Figure 1).

*S-phase fraction*

No correlation was found between SPF and the T- or pN category (Table III). Stage reduction and WHO grade 3 were more often found in the high SPF group than among tumours with low SPF ($P = 0.006$ and $P = 0.04$). Patients with high SPF tumours appeared to show a slightly better 5-year survival than those with low SPF tumours but this was not statistically significant ($P = 0.19$, Figure 2), unless neo-adjuvant chemotherapy had been given ($P < 0.05$, Figure 3). This latter finding is consistent with the observation that stage reduction was most often seen in high SPF tumours if the patients had received neo-adjuvant chemotherapy (Table IV). No difference of survival was observed between the SPF groups if no chemotherapy was given (Figure 4).

*Immunohistochemistry*

Forty-eight of 95 evaluable tumours (51%) stained for p53 protein (Table V). Comparable figures for HCG and c-*erb*B-2 were 12 and 9%, respectively. No correlation was found

**Figure 1** Cancer-related survival and DNA ploidy in 118 patients with T3/T2/T4a bladder cancer undergoing pre-operative radiotherapy and total cystectomy ± neo-adjuvant chemotherapy. ( ) number of patients within the subgroup.

**Figure 2** Cancer-related survival *and S-phase fraction (SPF)* in patients with T2/T3/T4a bladder cancer undergoing pre-operative radiotherapy and total cystectomy ± neo-adjuvant chemotherapy. ( ) Number of patients within the subgroup.

**Table II** DNA ploidy and clinical/histopathological parameters

| | | | *DNA non-diploid* | | |
|---|---|---|---|---|---|
| | *DNA diploid* | *DNA tetrapl.* | *DNA aneupl.* | *DNA non-dipl.* | *Total* |
| | *16*[a] | *30* | *72* | *102* | *118* |
| *T-category* | | | | | |
| T2 | 6 | 9 | 32 | 41 | 47 |
| T3/T4a | 10 | 21 | 40 | 61 | 71 |
| *Grade WHO* | | | | | |
| 1/2 | 7 | 6 | 16 | 22 | 29 |
| 3 | 9 | 24 | 56 | 80 | 89 |
| *Stage reduction* | | | | | |
| Yes ($P \leqslant$ T1) | 5 | 12 | 31 | 43 | 48 |
| No ($\geqslant$pT2) | 11 | 18 | 40 | 58 | 69 |
| Not evaluable[b] | | | 1 | 1 | 1 |
| *pN category* | | | | | |
| pN0 | 8 | 8 | 26 | 34 | 42 |
| $\geqslant$pN1 | 2 | 7 | 15 | 22 | 24 |
| Not evaluable | 6 | 15 | 31 | 46 | 52 |

[a]Number of patients in the group. [b]No laparatomy done.

**Figure 3** Cancer-related survival *and S-phase fraction (SPF)* in patients with T2/T3/T4a bladder cancer undergoing pre-operative radiotherapy and total cystectomy *with neo-adjuvant chemotherapy.* ( ) Number of patients within the subgroup.

**Figure 4** Cancer-related survival *and S-phase fraction (SPF)* in patients with T2/T3/T4a bladder cancer undergoing pre-operative radiotherapy and total cystectomy *without neo-adjuvant chemotherapy.* ( ) Number of patients within the subgroup.

between the DNA ploidy and the assessed immunohistological parameters. On the other hand, significantly more p53 protein positive tumours were observed in the low SFP group than the high SPF group ($P = 0.02$; Table VI). There was no statistically significant difference between the survival of patients with p53 protein positive tumours (54%) compared with those with p53 protein negative TCC (68%, $P = 0.40$).

### Discussion

The clinical management (cystectomy ± neo-adjuvant oncological treatment) and the overall outcome (60% 5-year disease-related survival) of the 118 patients from the present series are comparable to other published series presenting the results of total cystectomy in patients with operable muscle-invasive bladder cancer (Batata *et al.*, 1981; Scanlon *et al.*,

**Table III** S-phase fraction (SPF) and clinical/histopathological parameters

| | *Low SPF (57)*[a] | *High SPF (44)* | *Total (101)* |
|---|---|---|---|
| *T-category* | | | |
| T2 | 24 | 17 | 41 |
| T3/T4a | 33 | 27 | 60 |
| *WHO Grade*[b] | | | |
| 2 | 15 | 4 | 19 |
| 3 | 42 | 40 | 82 |
| *Stage reduction*[c,d] | | | |
| Yes | 18 | 22 | 40 |
| No | 39 | 21 | 60 |
| *pN category*[d] | | | |
| pN0 | 21 | 18 | 39 |
| ⩾pN1 | 8 | 11 | 19 |

[a]Number of patients within the group; [b]$P<0.001$; [c]$P<0.005$; [d]Evaluable patients only.

**Table IV** Chemotherapy, S-phase fraction (SPF) and stage reduction

| | *No chemotherapy* | | *Chemotherapy +* | |
|---|---|---|---|---|
| | *Low SPF (31)*[a] | *High SPF (22)* | *Low SPF (26)* | *High SPF (21)*[b] |
| *Stage reduction* | | | | |
| Yes | 7 | 7 | 11 | 15 |
| No | 24 | 15 | 15 | 6 |

[a]Number of evaluable patients in the group. [b]$P<0.08$ comparing both groups.

1983; Pagano *et al.*, 1988; Splinter *et al.*, 1992; Rintala *et al.*, 1992; Parson & Million, 1988). Our observations from the 118 patients, can thus be regarded generally applicable for patients with muscle-invasive bladder cancer scheduled for total cystectomy. A detailed analysis of the clinical parameters for all 186 patients will be an issue of a future report.

DNA ploidy determination in paraffin-embedded tumour material yields reliable and reproducible results in our hands. However, some inter-laboratory variation occurs in about 10% of the cases and has to be taken into account when comparing results from different groups (Coon *et al.*, 1988; Fosså *et al.*, 1992). We found that about 15% of our patients had DNA diploid tumours. This percentage is lower than reported in other series using paraffin-embedded material, including a previous report from our own group (Hug *et al.*, 1992; Malmstrøm *et al.*, 1989; Jacobsen *et al.*, 1987; Badalament *et al.*, 1990). The DNA diploidy rate of 15% is comparable to the figure of 10% reported by Wijkström and Tribukait (1990) when using fresh tumour samples.

**Table VI** S-phase fraction (SPF), p53 protein, HCG and c-*erb*B-2 protein

| | *Low SPF* | *High SPF* | *Total* |
|---|---|---|---|
| *p53 protein (65)*[a] | | | |
| Negative | 10 | 21 | 31 |
| Positive | 22 | 12 | 34 |
| + | 9 | 3 | 12 |
| + + | 6 | 2 | 8 |
| + + + | 7 | 7 | 14 |
| *HCG (61)*[a] | | | |
| Negative | 26 | 31 | 57 |
| Positive | 2 | 2 | 4 |
| *c-erbB-2 protein (60)*[a] | | | |
| Negative | 26 | 29 | 55 |
| Positive | 2 | 4 | 6 |

[a]Number of evaluable tumours.

DNA diploidy is not identical to the normal chromosomal number. Small quantitative DNA deviations may remain below the detection limit by FCM. Furthermore, DNA diploid and DNA near-diploid bladder tumours, as determined by FCM, may display cytogenetic deviations (Wijkström *et al.*, 1984; Coon *et al.*, 1986). In addition, from image cytometric studies it is obvious that some of the DNA diploid tumours contain small amounts of DNA non-diploid tumour cells, not detectable by FCM (Dawson *et al.*, 1990).

Our SPF values are within the ranges published by Lipponen *et al.* (1991) using similar techniques, and display significant differences between SPF values in DNA diploid and DNA non-diploid tumours. Evaluation of SPF by FCM yields, however, much less reliable and less reproducible observations, than DNA ploidy (Haag *et al.*, 1987) especially if paraffin-embedded tissue is used. The reasons for this variability may be randomly distributed (preparation technique, quality of the paraffin-embedded material) and are not easily compensated for. In addition, nuclear suspensions from sections contain sliced nuclei which contribute a systematic source of error when the SPF is determined. Some, but not all of these systematic errors can be accounted for by the computer program which calculates the different phases of the cell cycle. Using specially designed calculation programs for SPF acceptable correlations have been obtained between SPFs in fresh and paraffin-embedded tissue (Weaver *et al.*, 1990; Jacobsen *et al.*, 1992). In spite of all uncertainties in determination of SFP in cell suspensions from paraffin-embedded material SPF represents a clinical significant parameter in studies in human tumours (Merkel & McGuire, 1990). SPF determined by DNA FCM was therefore included in the present analysis.

The present homogeneous series of patients with muscle-invasive TCC confirms our and other authors' experience (Wijkström *et al.*, 1992; Jenkins *et al.*, 1990) on the very limited prognostic role of DNA ploidy in muscle-invasive bladder cancer. In a previous series DNA tetraploidy proved

**Table V** DNA ploidy, p53 protein, HCG and c-*erb*B-2 protein

| | *DNA diploid* | *DNA non-diploid* | | | *Total* |
|---|---|---|---|---|---|
| | | *DNA tetraploid* | *DNA aneuploid* | *DNA non-diploid* | |
| *p53 protein (95)*[a] | | | | | |
| Negative | 8 | 12 | 29 | 41 | 49 |
| Positive (all) | 4 | 12 | 30 | 42 | 46 |
| + | 2 | 6 | 13 | 19 | 21 |
| + + | 1 | 3 | 3 | 6 | 7 |
| + + + | 1 | 3 | 14 | 17 | 18 |
| *HCG (95)*[a] | | | | | |
| Negative | 12 | 19 | 53 | 72 | 84 |
| Positive | 0 | 6 | 5 | 11 | 11 |
| *c-*erb*B-2 protein (94)*[a] | | | | | |
| Negative | 11 | 23 | 52 | 75 | 86 |
| Positive | 1 | 2 | 5 | 7 | 8 |

[a]Number of evaluable tumours.

to be an independent prognostic parameter in patients receiving definitive radiotherapy (Jacobsen *et al.*, 1989). This finding was not supported by the present series consisting of operable bladder cancer. Our present results are in agreement with our previous findings (Jacobsen *et al.*, 1991) and a recent report by Hug *et al.* (1992) who confirmed that muscle-invasive bladder cancer with DNA diploid stemlines did not display a particularly favourable prognosis as might be expected.

Histological grade has in general been shown to correlate with proliferative activity in TCC, consistent with the present observations (Lipponen *et al.*, 1991; Tribukait *et al.*, 1982). Based on results from DNA flow cytometry in paraffin-embedded specimens Lipponen *et al.* (1991) and Shaaban *et al.* (1990) have suggested that a high SPF was correlated with the presence of regional lymph node metastases, a finding which is not supported by our results. In a preliminary report we have found a better prognosis for heterogeneously treated patients with advanced bladder cancer and high SPF when chemotherapy was given (Jacobsen *et al.*, 1992). This observation is confirmed in the present larger and homogeneous series where chemotherapeutically treated patients with high SPF tumours more often experienced stage reduction and had a better prognosis than those with low SPF. The reason may be that high SPF tumours due to their higher proliferation rate respond better to the combined neo-adjuvant onologic treatment (chemotherapy and radiotherapy) than do low SPF tumours. This correlation between SPF and stage reduction/prognosis suggests that patients with high SPF tumours should be offered pre-cystectomy treatment whereas such therapy is less indicated in patients with low SPF tumours.

The p53 suppressor gene participates in the cell cycle regulation (Levine *et al.*, 1991). The mutant p53 is assumed to inhibit normal p53 but there is also evidence for a direct oncogenic effect. Most studies suggest that p53 gene mutations arise relatively late in malignant progression. Mutations of the p53 gene have been reported in many human tumours (Porter *et al.*, 1992). Compared with other series reporting on frequency of the p53 gene mutations or its protein expression in bladder cancer (Sidransky *et al.*, 1991; Porter *et al.*, 1992) a relative high percentage (51%) of p53 protein positive bladder tumours was found in the present study. This might be due to the fact that all patients had muscle-invasive bladder cancer and more than 60% had deeply infiltrating TCC ($\geqslant$ T3). Fujimoto *et al.* (1992) have, for example, shown an increase of p53 gene mutations as bladder tumours become muscle-invasive.

We observed a high percentage of p53 positive tumours in the low SPF group, a relation which was maintained even when only p53 protein positive tumours graded as + + or + + + were considered. This observation is intriguing on the background of a recent report by Mørkve and Lærum (1991), who found a positive correlation between p53 reactivity and SPF when single cells from lung carcinoma were evaluated simultaneously by FCM. This discrepancy may be due to intra-tumoural heterogeneity and/or methodological differences between Mørkve and Lærum's observations and the present ones. Further experience in large homogeneous clinical series will be necessary to evaluate the clinical role of p53 in muscle-invasive bladder cancer.

Only about 10% of all bladder tumours in the present series were HCG and c-*erb*B-2 protein positive. These percentages are lower than reported previously by other groups (Wright *et al.*, 1990; Moriyama *et al.*, 1991; Coombs *et al.*, 1991; Jenkins *et al.*, 1990; Iles & Chard, 1991), but are consistent with another series from our group (Jacobsen *et al.*, 1990). We have not been able to confirm a correlation between DNA non-diploidy and c-*erb*B-2 protein immunoreactivity as indicated in our previous report on a more heterogeneous series (Berner *et al.*, 1992).

From the present series we summarise that DNA ploidy does not represent a prognostic parameter in patients who clinically are deemed to have operable muscle invasive TCC of the urinary bladder. However, patients with high SPF tumours seem to benefit from combined neo-adjuvant cytostatic and radiotherapeutic treatment more than those with low SPF tumours. About half of the bladder tumours were p53 protein positive, though the clinical relevance of this finding remains undetermined.

The study has been financially supported by grants from the EORTC GU Group and the Norwegian Cancer Society. The secretarial help of Liv Aagedal and Eldbjørg Gran is acknowledged.

### References

BADALAMENT, R.A., SMITH, J.J., FRANKLIN, G.L. & DRAGO, J.R. (1990). DNA ploidy analysis of invasive bladder cancer by flow cytometry. *In vivo*, **4**, 269–272.

BATATA, M.A., CHU, F.C.U., HILARIS, B.S., LEE, M.Z., VARESKO, R.W., LEE, H.S., VISETSIRI, E., KIM, Y.S., ONG, R. & WHITMORE, W.F. (1981). Preoperative whole pelvis versus true pelvis irradiation and/or cystectomy for bladder cancer. *Int. J. Radiat. Oncol. Biol. Phys.*, **7**, 1349–1355.

BERNER, AA., JACOBSEN, A.-B., FOSSÅ, S.D. & NESLAND, J.M. (1993). Expression of c-*erb*B-2 protein, neuron specific enolase and DNA cystectomy in locally advanced transitional cell carcinoma of the urinary bladder. *Histopathology*, **22**, 327–333.

COOMBS, L.M., PIGOTT, D.A., SWEENEY, E., PROCTOR, A.J., EYDMANN, M.E., PARKINSON, C. & KNOWLES, M.A. (1991). Amplification and overexpression of c-*erb*B-2 in transitional cell carcinoma of the urinary bladder. *Br. J. Cancer*, **63**, 601–608.

COON, J.S., SCHWARTZ, D., SUMMERS, J.L., MILLER, A.W. & WEINSTEIN, R.S. (1986). Flow cytometric analysis of deparaffinized nuclei in urinary bladder carcinoma. *Cancer*, **57**, 1594–1601.

COON, J.S., DEITCH, A.D., DE VERE WHITE, R.W., MELAMED, M.R., REEDER, J.E., WEINSTEIN, R.S., WERSTO, R.P. & WHEELESS, L.L. (1988). Inter-institutional variability in DNA flow cytometric analysis of tumors. *Cancer*, **61**, 126–130.

DAWSON, A.E., NORTON, J.AA. & WEINBERG, D.S. (1990). Comparative assessment of proliferation and DNA content in breast carcinoma by image analysis and flow cytometry. *Am. J. Pathol.*, **136**, 1115–1124.

FOSSÅ, S.D., BERNER, AA., WÆHRE, H., HEIDEN, THOMAS, HOLM JUUL, M.E., VAN DEN OUDEN, D., PETTERSEN, E.O., WANG, N. & TRIBUKAIT, B. (1992). DNA ploidy in cell nuclei from paraffin-embedded material-comparison of results from two laboratories. *Cytometry*, **13**, 395–403.

FOSSÅ, S.D., HARLAND, S.J., KAYE, S.B., RAGHAVAN, D., PARMAR, M.K.B., USCINSKA, B.M., WOOD, R. & THE MRC SUBGROUP IN ADVANCED BLADDER CANCER (ON BEHALF OF THE MRC UROLOGICAL WORKING PARTY). (1992). Initial combination chemotherapy with cisplatin, methotrexate and vinblastine in locally advanced transitional cell carcinoma-response rate and pitfalls. *Br. J. Urol.*, **70**, 161–168.

FUJIMOTO, K., YAMADA, Y., OKAJIMA, E., KAKIZOE, T., SUGIMURA, T. & TERADA, M. (1992). Frequent association of p53 gene mutation in invasive bladder carcinoma. *Cancer Res.*, **52**, 1393–1398.

GUSTAFSON, H., TRIBUKAIT, B. & ESPOSTI, P.L. (1982). DNA pattern, histological grade and multiplicity related to recurrence rate in superficial bladder tumours. *Scand. J. Urol. Nephrol.*, **16**, 135–139.

HAAG, D., FEICHTER, G., GOERTTLER, K. & KAUFMANN, M. (1987). Influence of systematic errors on the evaluation of the S phase portions from DNA distributions of solid tumours as shown for 328 breast carcinomas. *Cytometry*, **8**, 377–385.

HEDLEY, D.W., FRIEDLANDER, M.L., TAYLOR, I.W., RUGG, C.A. & MUSGROVE, E.A. (1983). Method for analysis of cellular DNA content of paraffin-embedded pathological material using flow cytometry. *J. Histochem. Cytochem.*, **31**, 1333–1335.

HIDDEMANN, W., SCHUMANN, J., ANDREEF, M., BARLOGIE, B., HERMAN, C.J., LEIF, R.C., MAYALL, B.H., MURPHY, R.F. & SANDBERG, A.A. (1984). Convention on nomenclature for DNA cytometry. *Cancer Genet. Cytogenet.*, **13**, 181–183.

HSU, S.M., RAINE, L. & FANGER, H. (1981). A comparative study of the peroxidase-antiperoxidase method and an avidin-biotin complex method for studying polypeptide hormones with radioimmunoassay antibodies. *Am. J. Clin. Pathol.*, **75**, 734–738.

HUG, E.B., DONNELLY, S.M., SHIPLEY, W.U., HENEY, N.M., KAUFMANN, D.S., PREFFER, F.I., SCHWARTZ, S.M., COLVIN, R.B. & ALTHAUSEN, A.F. (1992). Deoxyribonucleic acid flow cytometry in invasive bladder carcinoma: a possible predictor for successful bladder preservation following transurethral surgery and chemotherapy-radiotherapy. *J. Urol.*, **148**, 47–51.

ILES, R.K. & CHARD, T. (1991). Human chorionic gonadotropin expression by bladder cancers: biology and clinical potential. *J. Urol.*, **145**, 453–458.

JACOBSEN, A.-B., FOSSÅ, S.D., LUNDE, S., MELVIK, J.E. & PETTERSEN, E.O. (1987). Flow cytometric DNA measurements in paraffin-embedded bladder carcinoma tissue before and after precystectomy radiotherapy. *Radiotherapy & Oncol.*, **10**, 149–155.

JACOBSEN, A.-B., LUNDE, S., OUS, S., MELVIK, J.E., PETTERSEN, E.O., KAALHUS, O. & FOSSÅ, S.D. (1989). Multivariate analysis in 121 patients with T2/T3 bladder carcinomas treated with definitive radiotherapy with emphasis on flow cytometric DNA ploidy values. A study on paraffin-embedded tumour tissue. *Int. J. Radiat. Oncol. Biol. Phys.*, **17**, 923–929.

JACOBSEN, A.-B., NESLAND, J.N., FOSSÅ, S.D. & PETTERSEN, E.O. (1990). Human chorionic gonadotropin, neuron specific enolase and DNA flow cytometry in patients with high grade bladder carcinoma. *J. Urol.*, **143**, 706–709.

JACOBSEN, A.B., PETTERSEN, E.O., ÅMELLEM, Ø., BERNER, AA., OUS, S. & FOSSÅ, S.D. (1991). The prognostic significance of deoxyribonucleic acid flow cytometry in muscle invasive bladder carcinoma treated with preoperative irradiation and cystectomy. *J. Urol.*, **147**, 34–37.

JACOBSEN, A.-B., BERNER, AA., JUUL, M., OUS, S., PETTERSEN, E.O. & FOSSÅ, S.D. (1992). DNA flow cytometry and neo-adjuvant chemotherapy/radiotherapy in operable muscle invasive bladder carcinoma. A preliminary report. *Eur. Urol.* (in press).

JENKINS, B.J., MARTIN, J.E., BAITHUN, S.I., ZUK, R.J., OLIVER, R.T.D. & BLANDY, J.P. (1990). Prediction of response to radiotherapy in invasive bladder cancer. *Br. J. Urol.*, **65**, 345–348.

LEVINE, A.J., MOMAND, J. & FINLAY, C.A. (1991). The p53 tumour suppressor gene. *Nature*, **351**, 453–456.

LIPPONEN, P.K., ESKELINEN, M.J. & NORDLING, S. (1991). Progression and survival in transitional cell bladder cancer: a comparison of established prognostic factors, S-phase fraction and DNA ploidy. *Eur. J. Cancer*, **27**, 877–881.

MALMSTRØM, P.U., VASKO, J., WESTER, K., NORLÈN, B.J. & BUSCH, C. (1989). Flow cytometric analysis of DNA content of deparaffinized nuclei in urinary bladder carcinomas. Comparisons of different isolation methods and relation to histological grade and stage. *APMIS*, **97**, 811.

MASTERS, J.R.W., CAMPLEJOHN, R.S., CONSTANCE PARKINSON, M. & WOODHOSE, R.J. (1989). DNA ploidy and the prognosis of stage pT1 bladder cancer. *Br. J. Urol.*, **64**, 403–408.

MERKEL, D.E. & MCGUIRE, W.L. (1990). Ploidy, proliferative activity and prognosis. *Cancer*, **65**, 1194–1205.

MORIYAMA, M., AKIYAMA, T., YAMAMOTO, T., KAWAMOTO, T., KATO, T., SATO, K., WATANUKI, T., HIKAGE, T., KATSUTA, N. & MORI, S. (1991). Expression of c-*erb*B-2 gene product in urinary bladder cancer. *J. Urol.*, **145**, 423–437.

MØRKVE, O. & LAERUM, O.D. (1991). Flow cytometric measurement of p53 protein expression and DNA content in paraffin-embedded tissue from bronchial carcinomas. *Cytometry*, **12**, 438–444.

NORMING, U., TRIBUKAIT, B., GUSTAFSON, H., NYMAN, C.R., WANG, N. & WIJKSTRÖM, H. (1992). Deoxyribonucleic acid profile and tumor progression in primary carcinoma *in situ* of the bladder: a study of 63 patients with grade 3 lesions. *J. Urol.*, **147**, 11–15.

PAGANO, F., GUAZZIERI, S., ARTIBANI, W., PRAYE-GALETTI, T., MILANI, C., BASSI, P. & GARBEGLIO, A. (1988). Prognosis of bladder cancer. *Eur. Urol.*, **15**, 166–170.

PARSONS, J.T. & MILLION, R.R. (1988). Planned preoperative irradiation in the management of clinical stage B2-C (T3) bladder carcinoma. *Int. J. Radiat. Oncol. Biol. Phys.*, **14**, 797–810.

PORTER, P.L., GOWN, A.M., KRAMP, S. & COLTRERA, M.D. (1992). Widespread p53 overexpression in human malignant tumors. A immunohistological study using methacarn-fixed embedded tissue. *Am. J. Pathol.*, **140**, 145–153.

RINTALA, E., HANNISDAL, E., FOSSÅ, S.D., HELLSTEN, S. & SANDER, S. (1993). Neoadjuvant chemotherapy in bladder cancer: a randomized study. Nordic Cystectomy Trial I. *Scand. J. Urol. Nephrol.* (in press).

SCANLON, P.W., SCOTT, M. & SEGURA, J.W. (1983). A comparison of short-course, low-dose and long-course, high-dose preoperative radiation for carcinomas of the bladder. *Cancer*, **52**, 1153–1159.

SCHÜTTE, B., REYNDERS, M.M.J., BOSMAN, F.T. & BLIJHAM, G.H. (1985). Flow cytometric determination of DNA ploidy level in nuclei isolated from paraffin-embedded tissue. *Cytometry*, **6**, 26–30.

SHAABAN, A.A., TRIBUKAIT, B., EL-BEDEIWY, A.-F.A. & GHONEIM, M.A. (1990). Prediction of lymph node metastases in bladder carcinoma with deoxyribonucleic acid flow cytometry. *J. Urol.*, **144**, 884–887.

SIDRANSKY, D., VON ESCHENBACH, A., TSAI, Y.C., JONES, P., SUMMERHAYES, I., MARSHALL, F., PAUL, M., GREEN, P., HAMILTON, S.R., FROST, P. & VOGELSTEIN, B. (1991). Identification of p53 gene mutations in bladder cancers and urine samples. *Science*, **252**, 706–709.

SPLINTER, T.A.W., SCHER, H.I., DENIS, L., BUKOWSKI, R., SIMON, S., KLIMBERG, I., SOLOWAY, M., VOGELZANG, N.J., VAN TINTEREN, H., HERR, H. & EUROPEAN ORGANIZATION FOR RESEARCH ON TREATMENT OF CANCER – GENITOURINARY GROUP. (1992). The prognostic value of the pathological response to combination chemotherapy before cystectomy in patients with invasive bladder cancer. *J. Urol.*, **147**, 606–608.

STEEN, H.B. & LINDMO, T. (1979). Flow cytometry: a high-resolution instrument for everyone. *Science*, **204**, 403–404.

TRIBUKAIT, B., GUSTAFSON, H. & ESPOSTI, P.L. (1982). The significance of ploidy and proliferation in the clinical and biological evaluation of bladder tumours: a study of 100 untreated cases. *Br. J. Urol.*, **54**, 130–135.

TRIBUKAIT, B. (1987). Flow cytometry in assessing the clinical aggressiveness of genito-urinary neoplasms. *World J. Urol.*, **5**, 108–122.

UICC (UNION INTERNATIONALE CONTRE LE CANCER). (1978). In *TNM Classification of Malignant Tumours*. Third edition. International Union against Cancer. Harmer, M.H. (ed.). pp. 113–117, Geneva.

VINDELØV, L.L., CHRISTENSEN, I.J. & NISSEN, N.I. (1983). A detergent-trypsin method for the preparation of nuclei for flow cytometric DNA analysis. *Cytometry*, **3**, 323–327.

WEAVER, D.L., BAGWELL, C.B., HITCHCOX, S.A., WHETSTONE, S.D., BAKER, D.R., HERBERT, D.J. & JONES, M.A. (1990). Improved cytometric determination of proliferative activity (S-phase fraction) from paraffin-embedded tissue. *Am. J. Clin. Pathol.*, **94**, 576–584.

WIJKSTRØM, H. & TRIBUKAIT, B. (1990). Deoxyribonucleic acid flow cytometry in predicting response in radical radiotherapy of bladder cancer. *J. Urol.*, **144**, 646.

WIJKSTRØM, H., GRANBERG-ÖHMAN, I. & TRIBUKAIT, B. (1984). Chromosomal and DNA patterns in transitional cell bladder carcinoma. *Cancer*, **53**, 1718–1723.

WIJKSTRØM, H., NILSSON, B. & TRIBUKAIT, B. (1992). DNA analysis in predicting survival of irradiated patients with transitional cell carcinoma of bladder. *Br. J. Urol.*, **69**, 49–55.

WRIGHT, C., MELLON, K., NEAL, D.E., JOHNSTON, P., CORBETT, I.P. & HORNE, C.H.W. (1990). Expression of c-*erb*B-2 protein product in bladder cancer. *Br. J. Cancer*, **62**, 764–765.